# Genome organization is a major component of gene expression control in response to stress and during the cell division cycle in *trypanosomes*

**DOI:** 10.1098/rsob.120033

**Published:** 2012-04

**Authors:** S. Kelly, S. Kramer, A. Schwede, P. K. Maini, K. Gull, M. Carrington

**Affiliations:** 1 Department of Plant Sciences, University of Oxford, South Parks Road, Oxford OX1 3RB, UK; 2 Centre for Mathematical Biology, Mathematical Institute, University of Oxford, 24–29 St Giles’, OX1 3LB Oxford, UK; 3 Oxford Centre for Integrative Systems Biology, Department of Biochemistry, University of Oxford, South Parks Road, OX1 3QU Oxford, UK; 4 Department of Biochemistry, University of Cambridge, Tennis Court Road, Cambridge CB2 1QW, UK; 5 Sir William Dunn School of Pathology, University of Oxford, South Parks Road, Oxford OX1 3DR, UK

**Keywords:** evolution, gene expression, networks

## Abstract

The trypanosome genome is characterized by RNA polymerase II-driven polycistronic transcription of protein-coding genes. Ten to hundreds of genes are co-transcribed from a single promoter; thus, selective regulation of individual genes via initiation is impossible. However, selective responses to external stimuli occur and post-transcriptional mechanisms are thought to account for all temporal gene expression patterns. We show that genes encoding mRNAs that are differentially regulated during the heat-shock response are selectively positioned in polycistronic transcription units; downregulated genes are close to transcription initiation sites and upregulated genes are distant. We demonstrate that the position of a reporter gene within a transcription unit is sufficient to reproduce this effect. Analysis of gene ontology annotations reveals that positional bias is not restricted to stress–response genes and that there is a genome-wide organization based on proximity to transcription initiation sites. Furthermore, we show that the relative abundance of mRNAs at different time points in the cell division cycle is dependent on the location of the corresponding genes to transcription initiation sites. This work provides evidence that the genome in trypanosomes is organized to facilitate co-coordinated temporal control of gene expression in the absence of selective promoters.

## 2. Introduction

The trypanosomatids are a monophyletic group of unicellular eukaryotes [1,2]. The majority of characterized species are pathogenic, and collectively they inhabit a diverse range of hosts from coconut palms [3] to kangaroos [4], several causing globally important parasitic diseases of humans and livestock. In trypanosomatids, synthesis of mRNA by RNA polymerase II (RNAP II) occurs via polycistronic transcription [5]. Co-transcriptional processing to individual monocistronic mRNAs is mediated by trans-splicing of a 39-nucleotide-capped exon to the 5′ end of all protein-coding genes. Linked endonucleolytic cleavage and polyadenylation of the upstream mRNA complete the maturation process. This mechanism of gene expression is reflected in the structure and organization of the genome, where protein-coding genes are densely packed in polycistronically transcribed tandem arrays containing tens to hundreds of genes with greater than 50 per cent of the nucleotide sequence of the array present in mature mRNAs [6–8]. The RNAP II promoters driving transcription of these polycistronic arrays have yet to be characterized mechanistically, and selective initiation of transcription by RNAP II of particular gene arrays has not been demonstrated. Hence, it is thought that transcription is constitutive and thus the majority of gene expression regulation is mediated post-transcriptionally. Polycistronic transcription and *trans*-splicing are not unique to trypanosomatids, and are fundamental to the biology of many branches of the eukaryotic tree of life. In addition to trypanosomatids, many diverse eukaryotes (including appendicularia, ascidians, cnidarians, dinoflagellates, nematodes, platyhelminthes and rotifers) partially or entirely rely on this form of transcription for expression of their protein-coding genes [9–13].

The RNAP II promoters for protein-coding genes in trypanosomes are not fully characterized, but the location of the RNAP II transcription initiation sites for the procyclic developmental form of *Trypanosoma brucei* have been determined by precise transcriptional mapping [8]. A total of 191 RNAP II initiation sites were identified for protein-coding gene arrays, 129 were found at the 5′ end of the polycistronic gene arrays and 62 occurred within tandem gene arrays, indicating a more complicated pattern of transcription initiation than is apparent from analysis of the genome sequence alone. Despite a superficial similarity to bacterial operons, the identities of genes within transcription units in trypanosomes appear to lack functional clustering. There are a few exceptions, the most notable being the *Trypanosoma brucei* tubulin gene array on chromosome 1 that contains multiple repeats of the α- and β-tubulin genes [14,15]. However, it is unclear how this organization provides function to the cell, and in other trypanosomatids the α- and β- tubulin genes are in separate loci [14]. Some evidence has been provided that polycistrons can contain differentially expressed gene clusters [16]; however, caution should be exercised when analysing gene expression data from multi-locus high-copy-number gene families as the identity of the source locus cannot be unambiguously resolved. Despite a lack of obvious functional clustering, trypanosomatid genomes are highly syntenic [17]. On average, 70 per cent of the set of genes comprising each trypanosomatid genome share the same genomic context with other trypanosomatids [17,18]. This high degree of gene order conservation is astonishing given that the ancestors of trypanosomatids diverged hundreds of millions of years ago. A rationale for the strong conservation of gene order has yet to be identified.

In trypanosomatids, the absence of gene-specific promoters and the dependence on polycistronic transcription impacts on the ability of the cell to modulate gene expression in response to external stimuli. For example, on cellular stress in yeast and metazoa, there is an immediate response that operates through post-transcriptional mechanisms followed by a gene-specific transcriptional response. In the specific case of the heat-shock response, there is a rapid and selective inhibition of splicing [19,20]. A set of mRNAs, including those encoding heat-shock proteins (HSPs), escape the inhibition of splicing, and continue to be synthesized and exported. Other polyadenylated mRNAs are retained within the nucleus [21,22]. In addition, the half-life of some mRNAs, including HSP70, increases dramatically [23]. The subsequent transcriptional response is mediated by competition for HSP90 binding between heat-shock transcription factors and thermally sensitive proteins [24]. In trypanosomatids, the initial response to heat shock is similar. There is inhibition of splicing followed by a rapid decrease in levels of mRNA owing to increased turnover [25–27]. As in yeast and metazoa, a set of mRNAs, including those encoding HSPs, is excluded from this process and increases in relative abundance over the first hour of heat shock [27,28]. The lack of individual gene promoters means that a subsequent selective transcriptional response does not appear to be available and it has remained unclear how global patterns of gene expression are regulated in response to heat shock.

Here, an investigation of mechanisms compensating for the lack of a selective transcriptional response has led to the finding that there is a genome-wide functional organization of heat-shock-responsive genes. Rapidly downregulated genes tend to be located proximal to the transcription initiation site and upregulated genes tend to be distal. Furthermore, we demonstrate that spatial positioning of a reporter gene within a transcription unit is sufficient to alter temporal regulation of the corresponding mRNA's behaviour during heat shock. Extension of this observation to all genes with ascribed annotations reveals that selective positioning of groups of genes is not limited to heat-shock-responsive genes but is a general phenomenon of genome organization in *Trypanosoma brucei*.

## 3. Material and methods

### 3.1. Heat-shock expression data and genome position analysis

The GenBank file for the *Trypanosoma brucei* genome (Tbrucei_TriTrypDB-1.0.gff) was downloaded from TriTrypDB [29]. The locations of defined transcription initiation sites were retrieved from Kolev *et al*. [8]. The position for each gene relative to its nearest transcription initiation site in the correct direction was calculated. In cases where there was no identified transcription initiation site in the correct direction between a given gene and the end of the available sequence data, this sequence end was assumed to be the location of a putative transcription initiation site. Distances in nucleotides to transcription initiation sites were calculated based on the midpoint of each open reading frame. Pseudogenes, variant surface glycoproteins, expression-site-associated genes and genes with multiple genomic locations such as ‘retrotransposon hot spot protein’ were removed from all calculations. Heat-shock-induced changes in mRNA abundance were obtained from a previous study [27]. Fold change in mRNA abundance following heat shock was calculated from these microarray data. Only genes that were twofold or more differentially regulated in response to heat shock were selected for further analysis. All calculations, genome distance measurements and statistical tests were performed using Perl scripts.

### 3.2. Cells and reagents

*Trypanosoma brucei* Lister 427 procyclic forms were grown in SDM-79. All genetic manipulations used standard techniques. Cells were grown without antibiotic selection and below a density of 1 × 10^7^ cells ml^−1^ for two passages before any experiment. Measurements of mRNA half-lives were performed on cultures with cell densities between 4 and 7 × 10^6^ cells ml^−1^. For heat shock, 20 ml aliquots of cultures were placed in pre-warmed 30 ml glass centrifuge tubes in a 41°C water bath; the cultures took the first 4–5 min of the time course to reach 41°C. At selected time points, the tubes were removed from the water bath and cells pelleted by centrifugation at 3000*g* for 60 s. The pellet was resuspended in 1 ml serum-free medium; the cells were recovered by centrifugation in a microfuge for 15 s and resuspended in 50 μl of residual supernatant, and immediately frozen in an ethanol dry ice bath. In total, it took 3–4 min from water bath to freezing. RNA preparation and northern blotting were performed as previously described [27].

### 3.3. Gene ontology category analysis

To determine if particular groups of genes had biased locations within transcription units, the following analysis was performed. The complete list of genes with gene ontology (GO) term annotations was downloaded from TriTrypDB [29]. For each GO term category, the mean distance for the constituent group of genes to their respective transcription initiation sites was calculated. To determine whether these mean distances were higher or lower than expected if there was a random distribution of genes in the genome, a Monte Carlo resampling test was performed. For each GO term category containing 10 or more genes, the mean distance for the constituent genes to their nearest transcription initiation site was compared with the mean distance of randomly composed groups of genes of the same size. This process was repeated 10 000 times and the proportion of randomly selected groups that achieved a mean distance to transcription initiation sites of less than the GO term group was recorded.

To calculate an expected distribution for randomly composed GO term groups, the following procedure was performed. Each of the GO term categories containing 10 or more genes was randomly re-constituted from the set of genes with GO annotations. In cases where individual genes belonged to multiple GO categories, this relationship structure between GO categories was maintained by assigning the same randomly selected gene to all shared categories. The Monte Carlo distance test (described above) was then performed on this randomly resampled GO category dataset. An expected distribution was then calculated from 100 replicates of this randomization procedure.

### 3.4. Cell division cycle transcriptome analysis

The previously published cell-cycle-dependent transcriptome for early G1, late G1, S and G2/M phases of the cell division cycle of procyclic form *Trypanosoma brucei* were downloaded from Archer *et al*. [30]. The data were extracted and analysed in context of characterized transcription initiation sites, as above. A sliding window approach was taken to analyse these data. The window size was set to 20 kbp and was moved in 1 kbp steps in the direction of transcription away from the characterized transcription initiation sites. For each step, for each cell cycle stage, the mean mRNA abundance of all genes occurring with the 20 kb window (across all transcription units) was calculated. For each window position, the mean of all cell cycle stages was calculated and the log_2_ ratio of the individual cell cycle stage expression level relative to the mean was then taken.

## 4. Results

### 4.1. Genes differentially regulated in response to heat shock are not randomly distributed within polycistronic transcription units

**Figure 1.**
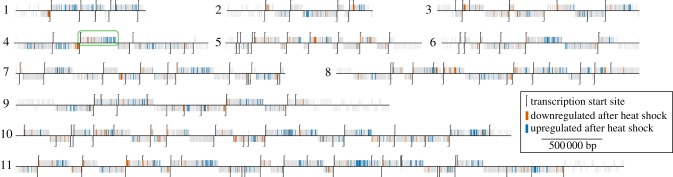
Genomic location of genes differentially regulated in response to heat shock. Each chromosome is depicted by a horizontal black line. Transcription initiation sites are indicated by vertical black lines. Genes encoding mRNAs that increase in abundance in response to heat shock by twofold or more are highlighted in blue and those encoding mRNAs that decrease by twofold or more are highlighted in red. Other genes are shown in grey. Genes above the chromosome line are transcribed left to right. Genes below the chromosome line are transcribed right to left. The green box highlights the polycistronic transcription on chromosome 4 unit selected for experimental testing.

A previous analysis of the heat-shock response in procyclic form *Trypanosoma brucei* identified 1058 mRNAs whose abundance changed in response to heat shock [27]. In the analysis presented here, the location of the heat-shock-responsive genes on each chromosome was determined. All 1058 mRNAs showing differential abundance in response to heat shock in the microarray experiment were selected. Three criteria were applied to the filter list. First, all mRNAs with a less than twofold response were removed to reduce the number of false positives arising from inaccuracies in the microarray data. Second, all mRNAs likely to be transcribed by RNA polymerase I (i.e. variant surface glycoprotein and expression site-associated genes) were removed. Third, mRNAs arising from dispersed multi-copy genes (i.e. GRESAG4 and ‘retrotransposon hotspot protein’) for which the microarray data cannot unambiguously distinguish the originating genes were also removed. The final list contained 211 mRNAs whose relative abundance decreased and 566 mRNAs whose relative abundance increased after heat shock (electronic supplementary material, file S1). Visual inspection of the distribution of the genes in this list relative to defined RNAPII transcription initiation sites [8] suggested that the genes corresponding to mRNAs whose relative abundance increased in response to heat shock were located further away from transcription initiation sites than those that decreased (figure 1; electronic supplementary material, file S1).

**Figure 2.**
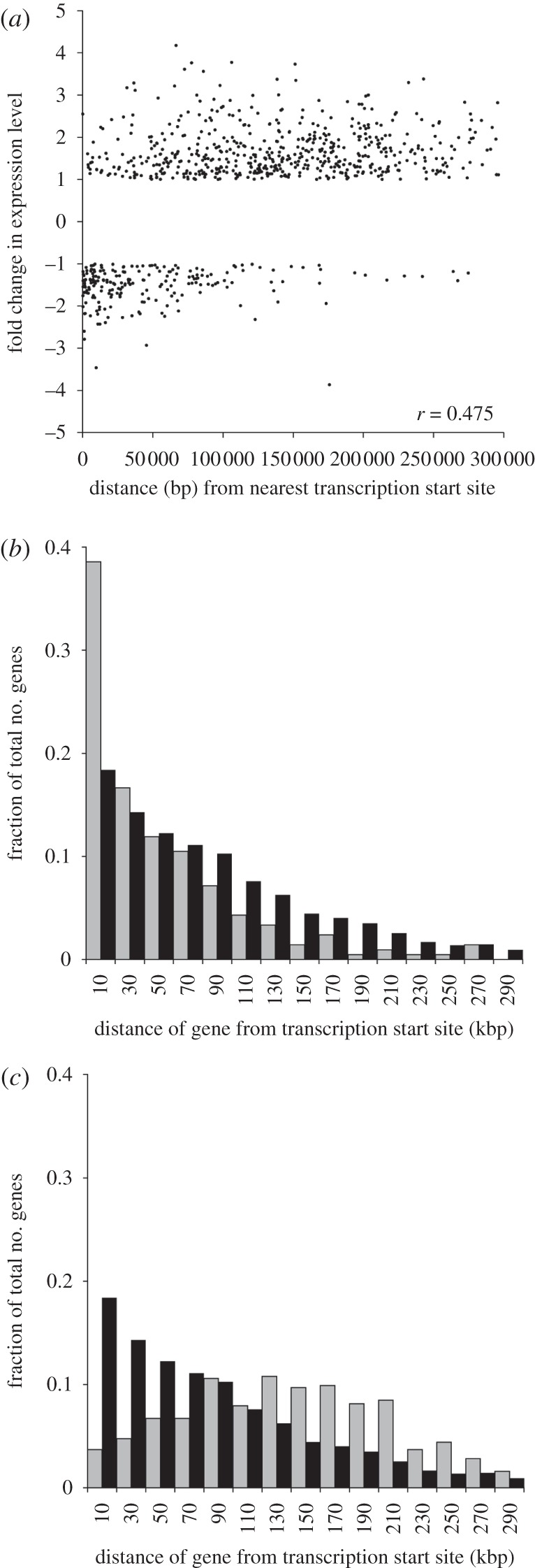
(*a*) Plot of fold change in mRNA abundance after heat-shock against distance of corresponding gene from nearest correct orientation transcription initiation site for mRNAs that were greater than twofold differentially regulated. (*b*) Histogram of proportion of genes at different distances from transcription initiation sites. Black bars indicate distribution of all genes in the genome. Grey bars indicate the distribution of genes whose mRNA abundance decreases by twofold or more on heat shock. (*c*) Histogram of proportion of genes at different distances relative to transcription initiation sites. Black bars indicate distribution of all genes in the genome. Grey bars indicate the distribution of genes whose mRNA abundance increases by twofold or more on heat shock.

To quantify and test this observation, the fold change in abundance of individual mRNAs was plotted against the distance of the cognate genes from their transcription initiation sites (figure 2*a*). This revealed that there was a significant positive correlation between these values, with Spearman's rank correlation coefficient *r* = 0.476, the probability of this being *p* < 0.0001 (Pearson product–moment correlation coefficient = 0.501, *p* < 0.0001). To determine whether this correlation reflects a bias in the relative position of heat-shock responsive genes, the location of these genes was interrogated in the context of the underlying distribution of all genes in the genome. More than 36 per cent of the genes whose mRNA abundance decreased following heat shock occur within 20 kbp of a transcription initiation site (figure 2*b*); this is more than twice the value expected if downregulated genes were distributed randomly in the genome (17.8%, figure 2*b*). In contrast to this, genes whose mRNA abundance increased in response to heat shock are under-represented near transcription initiation sites, with only 3 per cent of responsive genes occurring in the same interval (figure 2*c*). Moreover, genes encoding mRNAs that increased after heat shock are over-represented at distances greater than 120 kb from the nearest transcription initiation site (figure 2*c*). The mean distances for each group of genes are significantly different (all *p* < 0.0001, determined by Monte Carlo resampling of the data). While the full complement of genes that are necessary to mediate the heat-shock response is not yet defined, the two verified heat-shock-responsive HSPs [27] are both located near the ends of transcription units: both the tandem array of 10 HSP83 genes and the HSP70 (Tb11.01.3110) gene are located at larger-than-average distances from transcription start sites. This analysis provides evidence that there is a genome-wide functional positioning of genes within transcription units that contributes to the differential temporal response of mRNAs to stress, genes proximal to the initiation sites are downregulated following heat shock and genes distant to initiation sites are upregulated.

### 4.2. Genome positioning is sufficient to mediate differential regulation in response to heat shock

**Figure 3.**
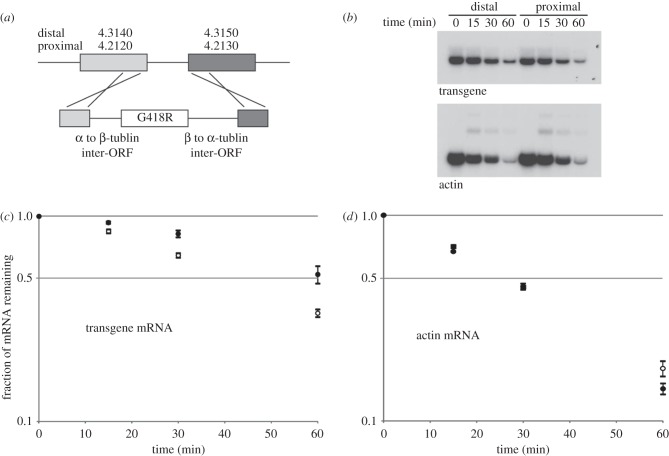
(*a*) Diagram to illustrate the strategy for integration of the reporter construct at different locations in the genome. Gene numbers are shown above target genes. G418R is the neomycin phosphotransferase open reading frame. (*b*) Northern blot of RNA samples prepared from procyclic form trypanosomes over a time course of heat shock at 41°C. The cell lines contained a reporter transgene at either approximately 284 kbp (distal) or approximately 8 kbp (proximal) from the transcription initiation site. The blots were probed to detect either the transgene mRNA or actin mRNA as a control for a normal heat-shock response. The results from one clone are shown. (*c*,*d*) Quantification of changes in mRNA abundance during heat shock for three independent clones for the distal and proximal (*c*) reporter gene and (*d*) actin mRNA control. In each case, the average for the three clones is shown, error bars indicate one standard error of the mean. The time shown is from transfer of the culture to 41°C; the culture reached this temperature between 4 and 5 min into the time course. Filled bars, distal; open bars, proximal.

The analysis above suggested that the distance of a gene from a transcription initiation site is sufficient to mediate a change in relative abundance of its corresponding mRNA during heat shock. To test this directly, a reporter transgene (encoding the neomycin resistance gene) was inserted at one or other of two positions in a transcription unit on chromosome 4 (figures 1 and 3*a*). The transcription unit on chromosome 4 from Tb927.4.2110 to Tb927.4.3190 was selected as it is long (approx. 294 kbp), it is clearly defined at each end by two inflection points in strand coding potential and it had no evidence for additional internal transcription initiation sites. The reporter constructs were designed to replace the sequence between two consecutive open reading frames (inter-ORF) with an α- to β-tubulin inter-ORF followed by the neomycin-resistant gene followed by a β- to α-tubulin inter-ORF (figure 3*a*). Tubulin inter-ORFs were chosen as the abundance of tubulin mRNAs are representative of the behaviour of an average mRNA following heat shock [27]. The transgene was inserted either between Tb927.4.3140 and Tb927.4.3150 (the midpoint between these two ORFs is approximately 284 kbp from the initiation of transcription) or between Tb927.4.2120 and Tb927.4.2130 (in this case, the midpoint is approximately 8 kbp from transcription initiation site). In either location, the transgene results in the expression of an identical mRNA with a β-tubulin 5′UTR followed by the neomycin phosphotransferase ORF followed by a β-tubulin 3′UTR. Independent cloned cell lines were isolated after growth in G418; none had an obvious growth defect, and no deleterious effect of transgene expression was observed (data not shown).

For both the distal and proximal position, the response of the transgene mRNA to heat shock in three independent clones was determined by northern blotting (figure 3*b*). At each of four time points, the mRNA level was quantified using a phosphorimager and normalized against total RNA (figure 3*c*). The rate of decrease was different for the distal and proximal transgene mRNAs. At all time points following heat shock, the level of the distal transgene mRNA was higher than that of the proximal (figure 3*c*). This was particularly apparent at 30 min, when the distal transgene mRNA had reduced to 82 per cent compared with 64 per cent from the proximal transgene. As a control, the steady-state level of the endogenous actin mRNA was determined in parallel for all time points. The decrease in abundance in all six cell lines was similar; there was a small difference between distal (14%) and proximal (18%) cell lines at 60 min, but this probably resulted from variations in estimating the low levels of actin mRNA at this time point. This analysis shows that the position of a gene from a transcription initiation site is sufficient to alter the relative abundance of the corresponding mRNA to heat shock. This position-dependent differential response provides a mechanism that contributes towards a differential abundance in the absence of a selective transcriptional response. mRNAs corresponding to genes distal to transcription initiation sites persist for longer following heat shock. Thus, as mRNAs corresponding to the proximal genes decrease more rapidly, this leads to a corresponding increase in the relative abundance of mRNAs corresponding to the distal genes.

### 4.3. Multiple categories of genes exhibit transcription unit positioning bias

**Figure 4.**
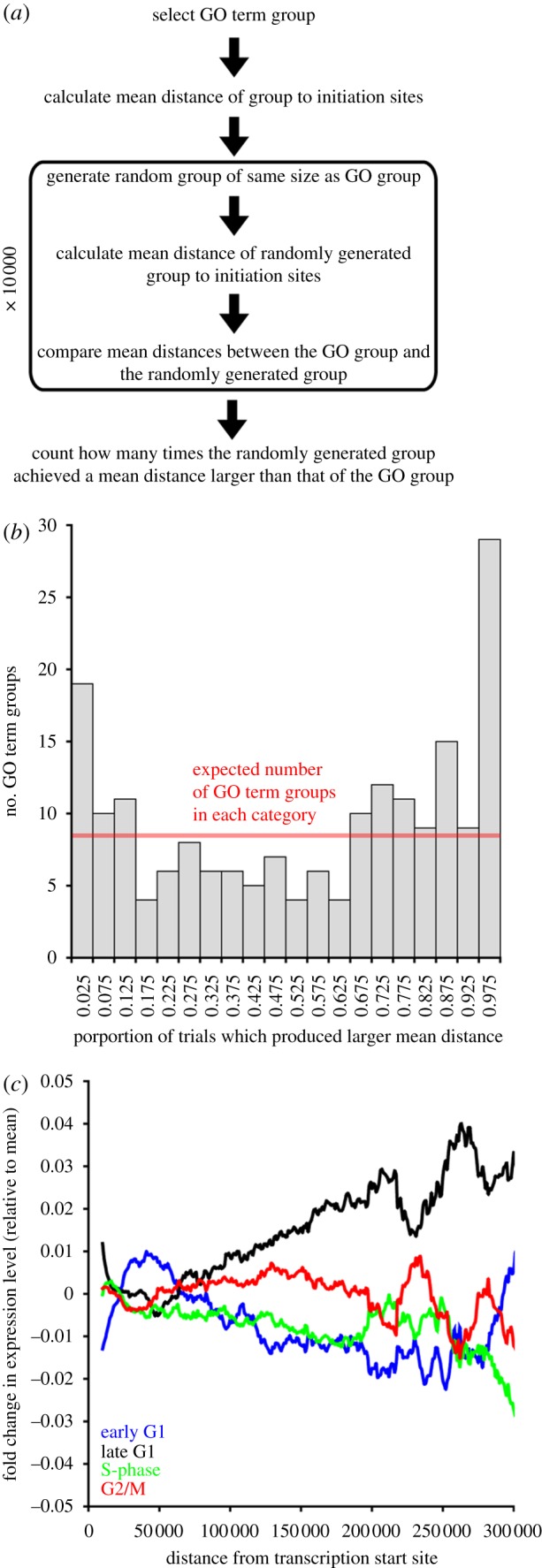
(*a*) Schematic cartoon describing the GO term group genome positioning analysis method. (*b*) Analysis of the distribution of gene ontology (GO) term groups with respect to transcription initiation sites. Grey bars indicate the number of GO term categories observed at each score level. Red line indicates the expected number of GO term categories at each score level if genes were randomly distributed in the genome. The red box encompasses the mean ± one standard error of the mean. (*c*) Plot of mean mRNA relative abundance within a 20 kb sliding window versus distance from transcription initiation site for four different cell cycle stages.

The above analyses showed that the position of a gene relative to a transcription initiation site is important for controlling the differential abundance of its mRNA in response to heat-shock-induced stress. To test whether other factors influence the location of genes within polycistronic units, two approaches were taken. First, other functional categories of genes were investigated to determine whether they showed positioning biases. GO annotations were used to group genes into categories. For each GO term category containing 10 or more genes, the mean and median distance of the genes to the nearest transcription initiation site was calculated (electronic supplementary material, file S2). The mean distance of this group was then compared with the mean distance of 10 000 randomly composed groups containing the same number of genes. The proportion of randomly selected groups that achieved a mean distance to transcription initiation sites of more than the GO term group was recorded (figure 4*a*; electronic supplementary material, file S2). For example, if 71 per cent of the randomly generated groups were further away from transcription start sites than the GO term group, then the GO term group would obtain a score of 0.71. This analysis showed that there are some GO term categories whose constituent genes are on average closer to transcription initiation sites than expected if genes were distributed randomly in the genome (figure 4*b* and table 1). Similarly, there are some GO term categories whose constituent genes are on average more distant from transcription start sites than expected if genes were distributed randomly in the genome (figure 4*b* and table 1). To control for discrepancies in gene density within transcription units and for differential transcription unit length, a further analysis was performed where a set of false GO term categories was reconstituted from randomly selected genes, each containing the same number of genes as the real GO term categories (see §3). The same distance-based analysis was completed as before and the procedure was repeated 100 times (figure 4*b*, red-shaded box). This shows that if genes were randomly distributed in the genome, an even distribution of GO term groups across transcription units would be observed.

**Table 1.** Gene ontology (GO) groups whose constituent genes are significantly enriched proximal to transcription initiation sites. Trials column shows the proportion of 10 000 trials that achieved a mean distance greater than the GO group. Grey shading indicates those groups for which less than 1 per cent of randomly selected groups achieved a smaller mean distance.

gene ontolody ID	GO term	number of members	mean distance (bp)	trials
GO:0006412	translation	225	64 454	1.0000
GO:0000786	nucleosome	50	46 456	1.0000
GO:0005509	calcium ion binding	76	53 025	1.0000
GO:0003735	structural constituent of ribosome	170	58 384	1.0000
GO:0019861	flagellum	23	29 276	1.0000
GO:0005840	ribosome	167	58 391	1.0000
GO:0006334	nucleosome assembly	54	48 557	1.0000
GO:0004722	protein serine/threonine phosphatase activity	26	48 084	0.9989
GO:0006928	cellular component movement	22	46 548	0.9986
GO:0022625	cytosolic large ribosomal subunit	16	41 906	0.9979
GO:0005886	plasma membrane	26	49 342	0.9970
GO:0051276	chromosome organization	50	60 721	0.9952
GO:0016469	proton-transporting two-sector ATPase complex	23	52 209	0.9928
GO:0007049	cell cycle	16	46 844	0.9926
GO:0005200	structural constituent of cytoskeleton	21	51 476	0.9919
GO:0005516	calmodulin binding	16	47 351	0.9909
GO:0044267	cellular protein metabolic process	12	43 843	0.9899
GO:0020037	haeme binding	21	53 098	0.9895
GO:0006470	protein dephosphorylation	42	61 173	0.9892
GO:0009405	pathogenesis	12	45 541	0.9862
GO:0015986	ATP synthesis-coupled proton transport	14	48 433	0.9830
GO:0008237	metallopeptidase activity	11	44 262	0.9825
GO:0042254	ribosome biogenesis	24	56 776	0.9807
GO:0004298	threonine-type endopeptidase activity	15	50 496	0.9797
GO:0005839	proteasome core complex	15	50 496	0.9793
GO:0004713	protein tyrosine kinase activity	150	73 351	0.9791
GO:0004197	cysteine-type endopeptidase activity	18	55 082	0.9724
GO:0004518	nuclease activity	12	49 843	0.9722
GO:0006812	cation transport	18	55 397	0.9664
GO:0009434	microtubule-based flagellum	17	55 802	0.9659
GO:0005622	intracellular	360	78 016	0.9654
GO:0016791	phosphatase activity	29	62 202	0.9639
GO:0000226	microtubule cytoskeleton organization	11	49 592	0.9618
GO:0004812	aminoacyl-tRNA ligase activity	24	60 712	0.9597
GO:0005783	endoplasmic reticulum	12	52 783	0.9546
GO:0005524	ATP binding	577	79 798	0.9543
GO:0015992	proton transport	21	60 391	0.9521
GO:0046034	ATP metabolic process	13	54 145	0.9516

For the 16 GO term categories that achieved a score of greater than or equal to 0.99 (i.e. ≤1% chance that randomly selected genes would obtain a mean distance less or equal to that observed for the GO category), four categories are concerned with translation (table 1). Interestingly, this set does not include genes encoding proteins involved in translation elongation and initiation, but rather the structural components of the ribosome (electronic supplementary material, file S2). In addition to the translation components, genes encoding components of the cytoskeleton and flagellum are also highly enriched proximal to transcription initiation sites (table 1).

Of the three GO term categories that achieved a score of 0.01 or less (i.e. ≤1% chance that randomly selected genes would obtain a mean distance greater or equal to that observed), two are concerned with transcription (table 2). Interestingly, within the 0.95 limit, all of the GO categories concerned with transcription were present. Hence, proteins of the transcription machinery are enriched at large distances from transcription initiation sites. The mean behaviour of these groups of genes in response to heat shock displays a highly significant positive correlation (Pearson *r* = 0.312, *p* < 0.00001, Spearman *r* = 0.314, *p* < 0.00001; electronic supplementary material, file S2), such that those mRNAs corresponding genes more distant from transcription initiation sites increase in abundance while those close to transcription initiation sites decrease. While the correlation is highly significant, it is low; therefore the requirements to respond to stress does not account for all the positioning biases of genes within transcription units observed here. Hence, this analysis suggests that differential positioning with respect to transcription initiation sites is also driven by other factors.

**Table 2.** GO groups whose constituent genes are significantly enriched distal to transcription initiation sites. Trials column shows the proportion of 10 000 trials that achieved a mean distance greater than the GO group. Grey shading indicates those groups for which less than 1 per cent of randomly selected groups achieved a greater mean distance.

gene ontolody ID	GO term	number of members	mean distance (bp)	trials
GO:0005275	amine transmembrane transporter activity	42	102 696	0.0462
GO:0005975	carbohydrate metabolic process	30	106 519	0.0450
GO:0006512	ubiquitin cycle	21	111 352	0.0446
GO:0030528	transcription regulator activity	14	118 847	0.0379
GO:0003678	DNA helicase activity	11	128 301	0.0241
GO:0016070	RNA metabolic process	14	123 344	0.0209
GO:0008168	methyltransferase activity	30	113 432	0.0189
GO:0016192	vesicle-mediated transport	65	104 361	0.0122
GO:0008565	protein transporter activity	17	125 736	0.0103
GO:0003700	sequence-specific DNA-binding transcription factor	16	127 754	0.0089
GO:0003899	DNA-directed RNA polymerase activity	37	118 739	0.0026
GO:0006464	protein modification process	66	114 931	0.0006

### 4.4. mRNA abundance during the cell division cycle is dependent on position relative to transcription initiation sites

The second approach was to analyse other expression datasets to determine whether mRNA abundance was related to gene position relative to transcription initiation site. The most marked effects were obtained from an analysis of mRNA abundance during the cell division cycle from a previous study [30]. Analysis of this data showed that mRNA abundance is dependent on position of the corresponding gene relative to the transcription initiation site at several points in the cell division cycle (figure 4*c*). In early G1, transcripts corresponding to genes positioned between 25 and 60 kbp from transcription initiation sites are relatively more abundant than during the remainder of the cell cycle (figure 4*c*). In late G1, relative mRNA abundance has a clear linear relationship with distance of the corresponding genes from transcription initiation sites (figure 4*c*). Here, the more distal a gene is from a transcription initiation site, the more abundant its mRNA will be in late G1 relative to other phases. In S-phase, mRNA abundance decreases with distance of the corresponding gene from the transcription initiation site. In G2/M, there is an increase in distal gene mRNA abundance (figure 4*c*). Taken together, this analysis shows that gene position has a pronounced effect on the relative abundance of mRNAs at different time points in the cell division cycle.

## 5. Discussion

The main findings in this paper are as follows. (i) Genes encoding mRNAs that increase during heat shock are not randomly distributed within transcription units, but tend to be located distal to the transcription initiation site. (ii) Genes encoding mRNAs that are downregulated rapidly on heat shock tend to be located close to a transcription initiation site. (iii) The location of a gene within a transcription unit is sufficient to modify the behaviour of the corresponding mRNA in response to heat shock. (iv) Positional bias is not limited to the heat-shock-responsive genes but rather multiple categories of genes display positional bias relative to transcription initiation sites. (v) Relative mRNA abundance in the different phases of the cell division cycle is related to the distance of the corresponding gene to transcription initiation sites. Taken together, these observations provide the first demonstration of multiple competing rules for gene location within polycistronic transcription units. They also show that spatial positioning contributes significantly to the temporal expression of genes and thus provide the first evidence for functional organization of the genome of *Trypanosoma brucei*.

The heat-shock response was initially investigated as the mRNA dynamics have been well documented. On heat shock, RNAP II transcription initiation is reduced and the half-life of many mRNAs is reduced [25,27]. In addition, there is a selective inhibition of the maturation of many mRNAs, but not HSP70 or HSP83 [28,31]. The effect of these changes is to reduce the total mRNA pool by 50 per cent in 1 h [27]. In this context, the data presented here are consistent with a model where, during heat shock, the initiation of transcription is reduced or stops, but elongation continues. There are no direct measurements of RNAP II transcription rates in trypanosomes but it is unlikely to differ greatly from the 4.3 kb min^−1^ determined in mammalian cells [32]. Therefore, as a cell enters heat shock, the reduction in initiation would affect genes proximal to the transcription initiation sites within a few minutes. However, genes distal to the initiation site would continue to be transcribed for longer as RNAP II complexes that had initiated before heat shock complete their transcription cycle. In the case of a gene approximately 250 kbp from a transcription initiation site, the journey time for RNAP II would be approximately 60 min and thus transcription of genes at these distances would continue for the first hour of heat shock. Any increased pausing of RNAP II would extend this further, thus providing a spatial mechanism to achieve specific temporal regulation.

By extending the analysis of position and function to include all genes with annotated GO terms, we have shown that there are specific biases in the location of genes involved in multiple different processes. Genes involved in translation, the cytoskeleton and the cell cycle are located proximal to transcription initiation sites, whereas genes involved in transcription and RNA processing are located distal to transcription initiation sites. We propose that the distal positioning of these genes may play a role in global regulation of transcription.

We have shown that relative transcript abundance during the cell division cycle is related to the position of the corresponding gene relative to transcription initiation sites. Hence, correct spatial positioning is critical for correct temporal expression in the cell division cycle. As genome position is not the sole factor controlling gene expression level, there are a number of possible reasons for the deviations from the linear responses observed in this analysis. One possibility is that they represent cell-cycle-stage-specific transcription initiation sites that have yet to be described. It will be interesting to see whether mapping the transcription start sites in a cell-cycle-dependent manner will facilitate the discovery of additional position-dependent effects. It is likely that the spatial effects we have observed here will play an important role in the regulation of gene expression in other groups of eukaryotes that use polycistronic transcription of tandem-arranged genes. It will also be interesting to see whether changes in the composition of the active polymerase complex [33] play a contributing role in these distance-dependent effects.

Is there evidence for a similar functional organization in the genomes of related species? A direct comparison with the genome of *Leishmania major* was not possible as the transcription initiation sites have not been systematically mapped. In *T. brucei*, 32 per cent (62/191) of transcription initiation sites occur without an inversion of stands with protein-coding potential, and this invalidates an analysis based on locating transcription initiation sites at strand inversion points. It will be interesting to see whether the functional organization is conserved once the transcription initiation sites are mapped in other kinetoplastids. Moreover, it will be interesting to see whether the same categories of genes display the same patterns of location.

In eukaryotes, temporal patterns of gene expression usually result from regulated initiation of transcription. In trypanosomes, this mechanism is absent and post-transcriptional mechanisms are thought to account for the majority of the regulation of gene expression. The genome-wide spatial organization of genes described here uncovers a new layer of expression-level control and facilitates an alternative mechanism to achieve temporal regulation of expression in the absence of specific initiation. Moreover, it provides specific evidence that a temporal programme of gene expression regulation has been hard-wired into the genome organization. Hence, a major component of temporal gene expression regulation in trypanosomes is achieved through spatial organization.

## Supplementary Material

Supplemental File 1

## Supplementary Material

Supplemental File 2

## References

[1] Hamilton PB Gibson WC Stevens JR 2007 Patterns of co-evolution between trypanosomes and their hosts deduced from ribosomal RNA and protein-coding gene phylogenies. Mol. Phylogenet. Evol. 44, 15–25 10.1016/j.ympev.2007.03.023 (doi:10.1016/j.ympev.2007.03.023) 17513135

[2] Simpson AG Gill EE Callahan HA Litaker RW Roger AJ 2004 Early evolution within kinetoplastids (Euglenozoa), and the late emergence of trypanosomatids. Protist 155, 407–422 10.1078/1434461042650389 (doi:10.1078/1434461042650389) 15648721

[3] Camargo EP 1999 Phytomonas and other trypanosomatid parasites of plants and fruit. Adv. Parasitol. 42, 29–112 10.1016/S0065-308X(08)60148-7 (doi:10.1016/S0065-308X(08)60148-7) 10050272

[4] Hamilton PB Stevens JR Gidley J Holz P Gibson WC 2005 A new lineage of trypanosomes from Australian vertebrates and terrestrial bloodsucking leeches (Haemadipsidae). Int. J. Parasitol. 35, 431–443 10.1016/j.ijpara.2004.12.005 (doi:10.1016/j.ijpara.2004.12.005) 15777919

[5] Van der Ploeg LH 1986 Discontinuous transcription and splicing in trypanosomes. Cell 47, 479–480 10.1016/0092-8674(86)90608-2 (doi:10.1016/0092-8674(86)90608-2) 3779833

[6] Wright JR Siegel TN Cross GA 2010 Histone H3 trimethylated at lysine 4 is enriched at probable transcription start sites in *Trypanosoma brucei*. Mol. Biochem. Parasitol. 172, 141–144 10.1016/j.molbiopara.2010.03.013 (doi:10.1016/j.molbiopara.2010.03.013) 20347883 PMC2875994

[7] Siegel TN Hekstra DR Kemp LE Figueiredo LM Lowell JE Fenyo D Wang X Dewell S Cross GA 2009 Four histone variants mark the boundaries of polycistronic transcription units in *Trypanosoma brucei*. Genes Dev. 23, 1063–1076 10.1101/gad.1790409 (doi:10.1101/gad.1790409) 19369410 PMC2682952

[8] Kolev NG Franklin JB Carmi S Shi H Michaeli S Tschudi C 2010 The transcriptome of the human pathogen *Trypanosoma brucei* at single-nucleotide resolution. PLoS Pathog. 6, e1001090 10.1371/journal.ppat.1001090 (doi:10.1371/journal.ppat.1001090) 20838601 PMC2936537

[9] LeBowitz JH Smith HQ Rusche L Beverley SM 1993 Coupling of poly(A) site selection and trans-splicing in *Leishmania*. Genes Dev. 7, 996–1007 10.1101/gad.7.6.996 (doi:10.1101/gad.7.6.996) 8504937

[10] Ullu E Matthews KR Tschudi C 1993 Temporal order of RNA-processing reactions in trypanosomes: rapid trans splicing precedes polyadenylation of newly synthesized tubulin transcripts. Mol. Cell. Biol. 13, 720–725 10.1128/MCB.13.1.720 (doi:10.1128/MCB.13.1.720) 8417363 PMC358950

[11] Mayer MG Floeter-Winter LM 2005 Pre-mRNA trans-splicing: from kinetoplastids to mammals, an easy language for life diversity. Mem. Inst. Oswaldo Cruz. 100, 501–513 10.1590/S0074-02762005000500010 (doi:10.1590/S0074-02762005000500010) 16184228

[12] Zhang H Hou Y Miranda L Campbell DA Sturm NR Gaasterland T Lin S 2007 Spliced leader RNA trans-splicing in dinoflagellates. Proc. Natl Acad. Sci. USA 104, 4618–4623 10.1073/pnas.0700258104 (doi:10.1073/pnas.0700258104) 17360573 PMC1838650

[13] Pouchkina-Stantcheva NN Tunnacliffe A 2005 Spliced leader RNA-mediated trans-splicing in phylum Rotifera. Mol. Biol. Evol. 22, 1482–1489 10.1093/molbev/msi139 (doi:10.1093/molbev/msi139) 15788744

[14] Jackson AP Vaughan S Gull K 2006 Evolution of tubulin gene arrays in Trypanosomatid parasites: genomic restructuring in *Leishmania*. BMC Genomics 7, 261 10.1186/1471-2164-7-261 (doi:10.1186/1471-2164-7-261) 17044946 PMC1621084

[15] Berriman M 2005 The genome of the African trypanosome *Trypanosoma brucei*. Science 309, 416–422 10.1126/science.1112642 (doi:10.1126/science.1112642) 16020726

[16] Veitch NJ Johnson PC Trivedi U Terry S Wildridge D MacLeod A 2010 Digital gene expression analysis of two life cycle stages of the human-infective parasite, *Trypanosoma brucei* *gambiense* reveals differentially expressed clusters of co-regulated genes. BMC Genomics 11, 124–126 10.1186/1471-2164-11-124 (doi:10.1186/1471-2164-11-124) 20175885 PMC2837033

[17] El-Sayed NM 2005 Comparative genomics of trypanosomatid parasitic protozoa. Science 309, 404–409 10.1126/science.1112181 (doi:10.1126/science.1112181) 16020724

[18] Daniels JP Gull K Wickstead B 2010 Cell biology of the trypanosome genome. Microbiol. Mol. Biol. Rev. 74, 552–569 10.1128/MMBR.00024-10 (doi:10.1128/MMBR.00024-10) 21119017 PMC3008170

[19] Yost HJ Lindquist S 1991 Heat shock proteins affect RNA processing during the heat shock response of *Saccharomyces cerevisiae*. Mol. Cell. Biol. 11, 1062–1068 1899282 10.1128/mcb.11.2.1062-1068.1991 PMC359779

[20] Shin C Feng Y Manley JL 2004 Dephosphorylated SRp38 acts as a splicing repressor in response to heat shock. Nature 427, 553–558 10.1038/nature02288 (doi:10.1038/nature02288) 14765198

[21] Saavedra C Tung KS Amberg DC Hopper AK Cole CN 1996 Regulation of mRNA export in response to stress in *Saccharomyces cerevisiae*. Genes Dev. 10, 1608–1620 10.1101/gad.10.13.1608 (doi:10.1101/gad.10.13.1608) 8682292

[22] Gallouzi IE Brennan CM Stenberg MG Swanson MS Eversole A Maizels N Steitz JA 2000 HuR binding to cytoplasmic mRNA is perturbed by heat shock. Proc. Natl Acad. Sci. USA 97, 3073–3078 10.1073/pnas.97.7.3073 (doi:10.1073/pnas.97.7.3073) 10737787 PMC16194

[23] Theodorakis NG Morimoto RI 1987 Posttranscriptional regulation of hsp70 expression in human cells: effects of heat shock, inhibition of protein synthesis, and adenovirus infection on translation and mRNA stability. Mol. Cell. Biol. 7, 4357–4368 10.1128/MCB.7.12.4357 (doi:10.1128/MCB.7.12.4357) 3437893 PMC368119

[24] Zou J Guo Y Guettouche T Smith DF Voellmy R 1998 Repression of heat shock transcription factor HSF1 activation by HSP90 (HSP90 complex) that forms a stress-sensitive complex with HSF1. Cell 94, 471–480 10.1016/S0092-8674(00)81588-3 (doi:10.1016/S0092-8674(00)81588-3) 9727490

[25] Muhich ML Boothroyd JC 1988 Polycistronic transcripts in trypanosomes and their accumulation during heat shock: evidence for a precursor role in mRNA synthesis. Mol. Cell. Biol. 8, 3837–3846 10.1128/MCB.8.9.3837 (doi:10.1128/MCB.8.9.3837) 3221866 PMC365442

[26] Schwede A Kramer S Carrington M 2011 How do trypanosomes change gene expression in response to the environment? Protoplasma 249, 223–238 10.1007/s00709-011-0282-5 (doi:10.1007/s00709-011-0282-5) 21594757 PMC3305869

[27] Kramer S Queiroz R Ellis L Webb H Hoheisel JD Clayton C Carrington M 2008 Heat shock causes a decrease in polysomes and the appearance of stress granules in trypanosomes independently of eIF2(alpha) phosphorylation at Thr169. J. Cell. Sci. 121, 3002–3014 10.1242/jcs.031823 (doi:10.1242/jcs.031823) 18713834 PMC2871294

[28] Muhich ML Hsu MP Boothroyd JC 1989 Heat-shock disruption of trans-splicing in trypanosomes: effect on Hsp70, Hsp85 and tubulin mRNA synthesis. Gene 82, 169–175 10.1016/0378-1119(89)90042-5 (doi:10.1016/0378-1119(89)90042-5) 2684772

[29] Aslett M 2010 TriTrypDB: a functional genomic resource for the Trypanosomatidae. Nucleic Acids Res. 38, D457–D462 10.1093/nar/gkp851 (doi:10.1093/nar/gkp851) 19843604 PMC2808979

[30] Archer SK Inchaustegui D Queiroz R Clayton C 2011 The cell cycle regulated transcriptome of *Trypanosoma brucei*. PLoS ONE 6, e18425 10.1371/journal.pone.0018425 (doi:10.1371/journal.pone.0018425) 21483801 PMC3069104

[31] Muhich ML Boothroyd JC 1989 Synthesis of trypanosome hsp70 mRNA is resistant to disruption of trans-splicing by heat shock. J. Biol. Chem. 264, 7107–7110 2708359

[32] Darzacq X Shav-Tal Y de Turris V Brody Y Shenoy SM Phair RD Singer RH 2007 *In vivo* dynamics of RNA polymerase II transcription. Nat. Struct. Mol. Biol. 14, 796–806 10.1038/nsmb1280 (doi:10.1038/nsmb1280) 17676063 PMC4942130

[33] Harel-Sharvit L Eldad N Haimovich G Barkai O Duek L Choder M 2010 RNA polymerase II subunits link transcription and mRNA decay to translation. Cell 143, 552–563 10.1016/j.cell.2010.10.033 (doi:10.1016/j.cell.2010.10.033) 21074047

